# Uncemented science at its best! What makes a paper successful, meaning in this case much cited?

**DOI:** 10.1080/17453674.2020.1763566

**Published:** 2020-05-14

**Authors:** Anne Lübbeke

**Affiliations:** Division of Orthopaedic Surgery and Traumatology, Geneva University Hospitals, Switzerland;; Nuffield Department of Orthopaedics, Rheumatology and Musculoskeletal Sciences, University of Oxford, UK

It has been suggested that a short title, as here, is one of the keys to success. More seriously, the paper by Hailer, Garellick and Kärrholm (2010) addressed a relevant, complex and controversial question of high clinical utility: the surgeon’s choice of fixation of a primary total hip arthroplasty and the influence of this decision on short- to mid-term implant survivorship and revision causes. And although the evidence has evolved, the topic is still much discussed today. This is illustrated by the fact that the paper has been cited more than 200 times, with an average citation of over 20 each year, and no sign of declining interest in the last years.

The authors based their study on a very large number of operations (more than 170,000 primary THAs operated 1992–2007) from the Swedish Hip Arthroplasty Register and on a robust data collection and analysis methodology. The introduction and discussion sections were comprehensive, multifaceted, thought provoking and well written. As always in clinical research, studying the influence of a specific factor/exposure on an outcome has to take into account its multifactorial context, and has to be aware of the fact that it is conducted in a given time and place. Here the influence of implant fixation on prosthesis longevity was evaluated in the interplay with the individual prosthesis brand(s), the surgeon’s experience and patient characteristics. Moreover, their outcome comparison took into account that choice of fixation might influence short-, mid-, long-term survival differently and might lead to different revision cause patterns. The authors found that THAs with all cemented fixation had overall a much better survival (all-cause revision) at 2, 10 and 15 years than all uncemented fixation, with 10-year survival of 85% for uncemented and 94% for cemented THAs. No age group benefitted from the use of uncemented fixation. Nonetheless, the study did not conclude with a “one size fits all” message. Instead they pointed out that uncemented cups were “the Achilles’ tendon of uncemented THA” and that uncemented stems performed better overall than cemented stems. However, use of uncemented stems was associated with an eight times greater risk of stem revision for periprosthetic fracture in the first two years after surgery.

Others have studied the same question in the following years. Among them I will point out two publications from the same place (Scandinavia), but different time periods. The first study (Mäkelä et al. 2014) evaluated 347,899 primary THAs operated 1995–2011 from the Nordic Arthroplasty Register Association and found a lower overall 10-year survival (all-cause revision) of 91.4–93.5% for all uncemented vs. 93.4–95.0% for all cemented THAs with an improvement in survival for both types of fixation from the late nineties to the early 2000 years. Looking at age, the survival disadvantage of all uncemented fixation was present in patients aged 65 years or older, but not in younger patients. Periprosthetic fracture was a more common cause of revision after uncemented (27%) than after cemented fixation (4%).

The second study (Dale et al. 2020) evaluated 66,995 primary THAs operated in Norway 2005–2017 overall and by strata of fixation, age and sex. The 10-year survival (all-cause revision) was 94% with all uncemented and 95% with all cemented fixation. The authors stated: “We found good overall survival for common, contemporary, well-documented primary THAs regardless of fixation method: cemented, uncemented, reverse hybrid, or hybrid fixation. However, uncemented THAs had a slightly higher overall risk of revision compared with cemented THAs. This difference was mainly caused by an increased risk of periprosthetic fracture and dislocation after uncemented THA, in particular when used in elderly women.”

Summarizing the development from the early nineties until most recently: First, 10-year survival of primary THA with all uncemented fixation has improved since the study by Hailer et al. Second, there are still slightly more revisions overall with all uncemented vs. all cemented fixation, mainly happening during the first year(s). Third, the well-known, repeatedly highlighted issue of a much higher incidence of – especially intraoperative – periprosthetic fractures with the use of uncemented stems has not been solved (Abdel et al. 2016, Dale et al. 2020, Hailer et al. 2010, Mäkelä et al. 2014). This complication has serious consequences including substantially higher mortality and stem revision rates early on and up to 10 years following the uncemented primary THA (Lamb et al. 2019a). Solutions have been proposed consisting among others of avoidance of uncemented fixation in elderly patients, and in particular in women over 75 years of age (Abdel et al. 2016, Dale et al. 2020, Lamb et al. 2019b).

At a time when the personalized/stratified medicine approach for improved patient-centred care is on many lips, here is an area to apply it to. The paper by Hailer, Garellick, and Kärrholm paved the way.

Anne Lübbeke *Division of Orthopaedic Surgery and Traumatology, Geneva University Hospitals, Switzerland* *Nuffield Department of Orthopaedics, Rheumatology and Musculoskeletal Sciences, University of Oxford, UK* *E-mail:*anne.lubbekewolff@hcuge.ch
